# Sarcoidosis with marked necrosis in enlarged lymph nodes mimics mycobacterial infection: a case report

**DOI:** 10.1186/s13256-021-02797-3

**Published:** 2021-04-18

**Authors:** Yosuke Miyashita, Munechika Hara, Shin-ichiro Iwakami, Hironari Matsuda, Naoko Iwakami, Kazuhisa Takahashi

**Affiliations:** 1 Department of Respiratory Medicine, Juntendo University Shizuoka Hospital, 1129 Nagaoka, Izunokuni, Shizuoka 410-2295 Japan; 2 Department of Respiratory Medicine, Juntendo University Graduate School of Medicine, 2-1-1 Hongo, Bunkyo-Ku, Tokyo, 113-8421 Japan

**Keywords:** Epithelioid cell granuloma, Mycobacterial infection, Necrosis, Necrotizing sarcoid granulomatosis, Sarcoidosis

## Abstract

**Background:**

Sarcoidosis is pathologically characterized by the formation of non-necrotizing epithelioid cell granulomas. However, pathological findings of patients with sarcoidosis have rarely revealed necrosis. We report here on a patient with sarcoidosis which needed to be distinguished from infectious disease because of marked necrosis in the lymph nodes.

**Case presentation:**

A 46-year-old Japanese woman was referred to our hospital due to a dry cough and appetite loss. A chest X-ray and computed tomography revealed markedly enlarged mediastinal and hilar lymph nodes and hepatosplenomegaly. Surgical biopsy of these lymph nodes was performed in order to make a diagnosis. Pathological findings revealed epithelioid cell granuloma with marked necrosis that suggested infectious etiology such as mycobacterial and fungal infections. In addition to the pathological findings, immunoglobulin A (IgA) antibody for *Mycobacterium avium* complex (MAC), enlargement of lymph nodes and hepatosplenomegaly indicated disseminated MAC, while sarcoidosis was considered as another important differential diagnosis according to elevated angiotensin-converting enzyme, soluble interleukin-2 receptor and uveitis. While waiting for the results of the cultures of acid-fast bacilli, the symptoms of cough and consumption had worsened, and initiation of therapy was required before the confirmed diagnosis. The therapy for MAC was initiated because it was feared that immunosuppressive therapy containing corticosteroid for sarcoidosis could worsen the patient’s condition if MAC infection was the main etiology. However, the treatment for MAC was not effective, and it was clarified that no acid-fast bacilli were cultured in the liquid culture medium, so the diagnosis was corrected to sarcoidosis after reconsideration of clinical and pathological findings. Prednisolone (30 mg/day) was administered orally, and the patient’s symptoms and radiological findings improved.

**Conclusion:**

Sarcoidosis must be considered even if pathological findings reveal marked necrosis, because rare cases of sarcoidosis exhibit extensive necrosis in lymph nodes. It is extremely important to carefully examine the clinical and pathological findings through discussion with the examining pathologist to reach the correct diagnosis.

## Background

Sarcoidosis is a systemic disease of unknown etiology, frequently affecting the lung and lymphatic systems, pathologically characterized by the formation of non-necrotizing epithelioid cell granulomas [1]. Although the granulomas of sarcoidosis occasionally exhibit focal necrosis, infectious etiology is usually suspected in cases with granulomas that contain a greater degree of necrosis [1]. Sarcoidosis and infectious disease such as mycobacterial and fungal infections have completely different treatment methods. Careful differentiation based on a comprehensive review of clinical, laboratory and pathological findings is extremely important.

Here, we report on a patient with sarcoidosis which needed to be distinguished from infectious disease because of marked necrosis in the lymph nodes.

## Case presentation

**Fig. 1:**
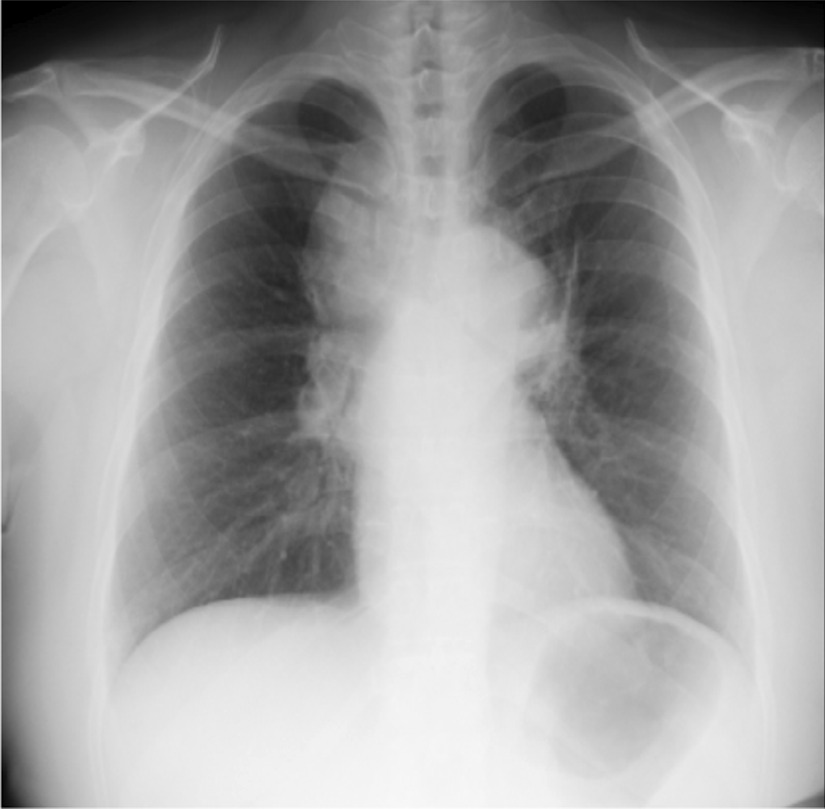
Chest X-ray showing an upper mediastinal widening

**Fig. 2:**
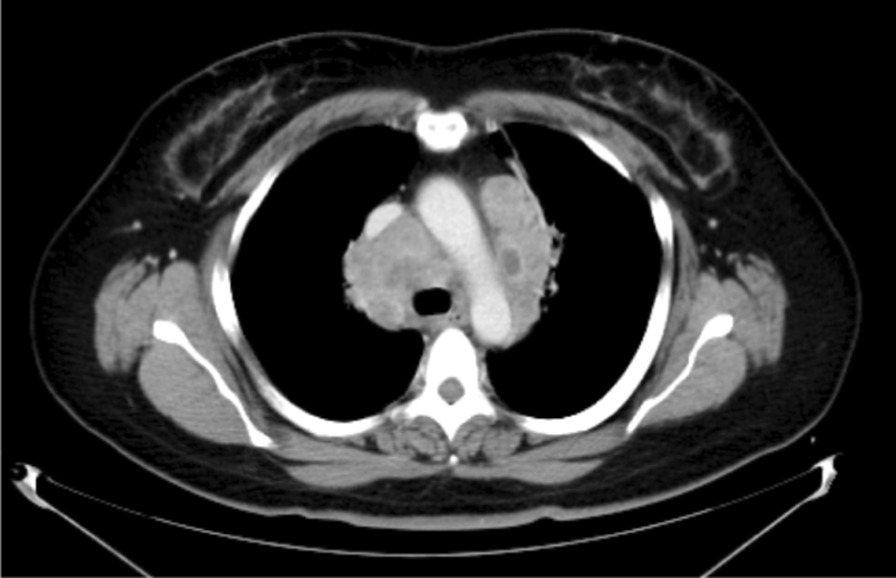
Thoracic computed tomography with contrast enhancement showing mediastinal lymph node swelling which contained low-density areas

A 46-year-old Japanese woman with no smoking history was referred to our hospital with a dry cough that had been worsening for a month. The patient had a slight loss of appetite, and no weight loss. There was no medical history, no family history of lung disease and no allergies. She had one gravidity and one parity, and had been a homemaker since her twenties, living with her husband and one child. Her socioeconomic status was not low. She drank socially. Her vital signs were within the normal range: body temperature, 36.8 °C; blood pressure, 130/88 mm Hg; pulse rate, 88 beats per minute. Heart sounds were normal without murmurs, and breath sounds were clear on auscultation. Her abdomen was soft and flat. Superficial lymph nodes were not palpable and there were no skin lesions. There were no significant neurological findings. The patient had uveitis in both anterior eyes as a result of medical examination by an ophthalmologist. Complete blood counts were normal: white blood cell count, 5300/µL; hemoglobin, 14.7g/dL; platelet count, 201,000/µL. Liver and renal function were both normal, C-reactive protein was 0.76 mg/dL, serum calcium was 9.7 mg/dL, angiotensin-converting enzyme (ACE) was elevated at 35.1 U/L, soluble interleukin-2 receptor (sIL-2R) was elevated at 1693 U/mL, and antinuclear antibody was within normal range. Urinalysis showed no special findings. Blood and sputum cultures were negative. A *Mycobacterium tuberculosis*-specific interferon-γ release assay was negative, but the serum anti-glycopeptidolipid core immunoglobulin A (IgA) antibody for *Mycobacterium avium* complex (MAC) was positive. *Aspergillus*-galactomannan antigen, beta-d-glucan, *Cryptococcus neoformans* antigen, perinuclear antineutrophil cytoplasmic antibody, and cytoplasmic antineutrophil cytoplasmic antibody were within normal range. Anti-human immunodeficiency virus antibody was negative. The chest X-ray showed an upper mediastinal widening (Fig. 1). Contrast-enhanced thoracic computed tomography (CT) showed swelling of the mediastinal and hilar lymph nodes, which contained low-density areas and hepatosplenomegaly (Fig. 2).

**Fig. 3:**
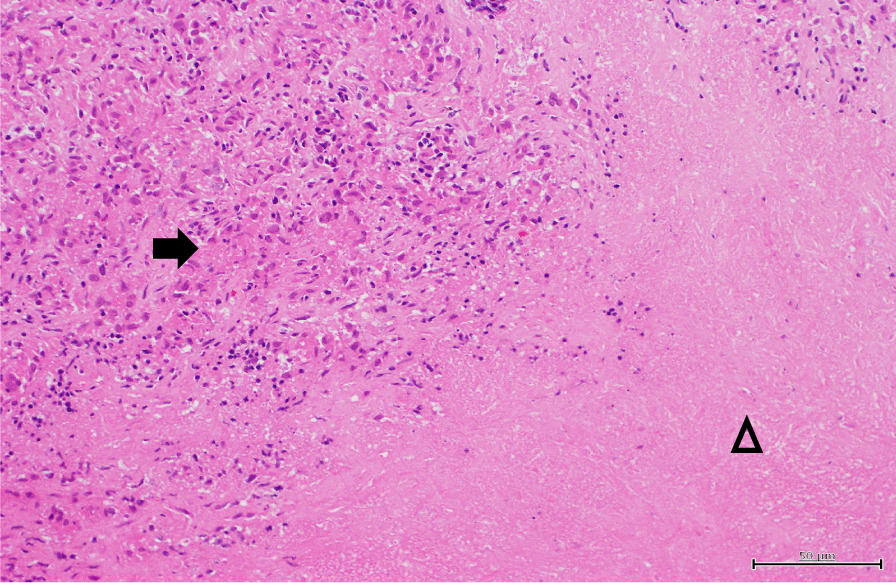
Histopathological findings of the specimen obtained from the mediastinal lymph nodes showing epithelioid cell granuloma (black arrow) and necrosis (triangle) (hematoxylin and eosin staining, ×200)

A fiber bronchoscopy was performed. The bifurcation of the trachea was dull and the bronchial mucosa was erythrogenic and edematous. Subsequently, endobronchial ultrasound-guided transbronchial needle aspiration was performed under adequate conscious sedation to obtain a specimen from the mediastinal lymph node. However, a sufficiently large specimen for evaluation could not be obtained due to the patient’s severe cough. She underwent surgical lymph node biopsy in order to differentiate malignancy, mycobacterial infection or sarcoidosis. The specimen obtained from the mediastinal lymph nodes revealed epithelioid cell granuloma with marked necrosis (Fig. 3). Ziehl–Neelsen staining and polymerase chain reaction of *Mycobacterium tuberculosis* and MAC using the obtained specimen were negative. Based on the pathological findings of epithelioid cell granuloma, mycobacterial infection, fungi, vasculitis and sarcoidosis were considered as differential diagnosis. The possibility of *Mycobacterium tuberculosis*, fungi or vasculitis was considered low because *Mycobacterium tuberculosis*-specific interferon-γ release assay, *Aspergillus*-galactomannan antigen, beta-d-glucan, *Cryptococcus neoformans* antigen, perinuclear antineutrophil cytoplasmic antibody, and cytoplasmic antineutrophil cytoplasmic antibody were negative. While elevated ACE, sIL-2R and uveitis suggested sarcoidosis, marked necrosis of epithelioid cell granuloma and IgA antibody for MAC strongly indicated MAC infection, especially disseminated MAC according to swelling of the mediastinal and hilar lymphadenopathy and hepatosplenomegaly. While waiting for the results of the cultures of acid-fast bacilli, the symptoms of cough and consumption had worsened, and initiation of therapy was required before the confirmed diagnosis. Oral clarithromycin (600 mg/day), rifampicin (450 mg/day) and ethambutol (750 mg/day) as therapy for MAC were initiated for 3 weeks, because it was feared that immunosuppressive therapy containing corticosteroid for sarcoidosis could worsen the patient’s condition if MAC infection was the main etiology. Clarithromycin was started at a reduced dose because of her appetite loss and the concern regarding tolerability for triple-drug combination therapy for MAC, and dose escalation was planned if it was well tolerated.

**Fig. 4:**
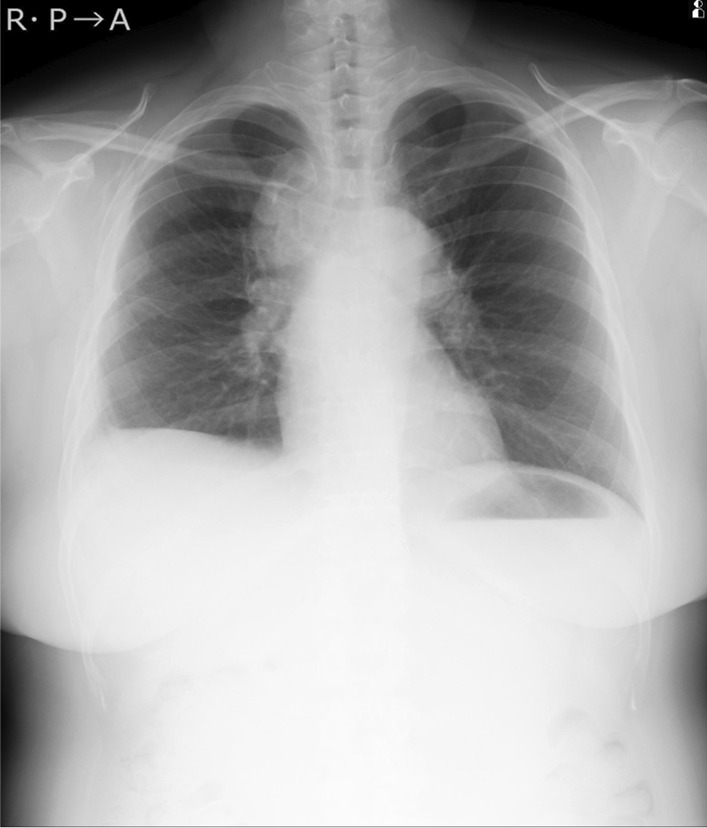
Chest X-ray at 2 weeks after administration of steroid

**Fig. 5:**
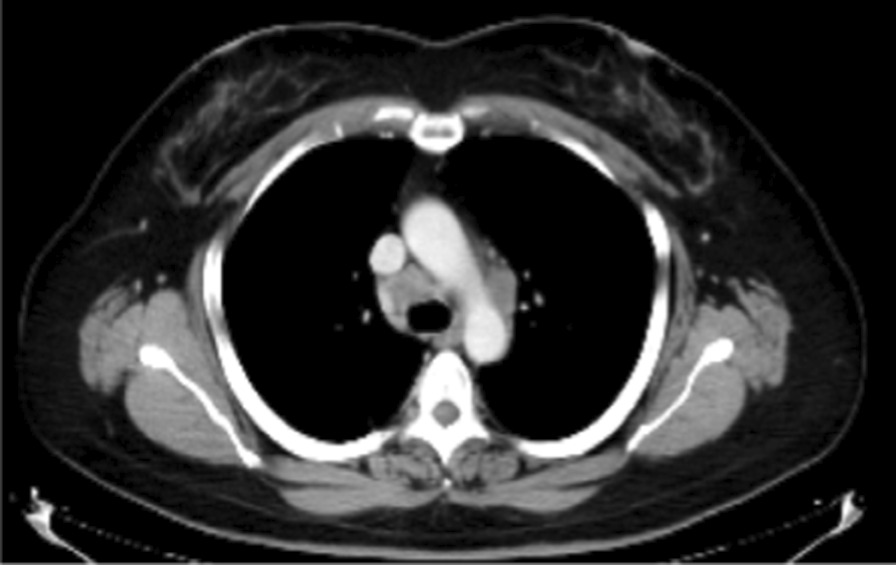
Thoracic computed tomography with contrast enhancement at 2 weeks after administration of steroid showing reduced mediastinal lymph nodes in size

**Fig. 6:**
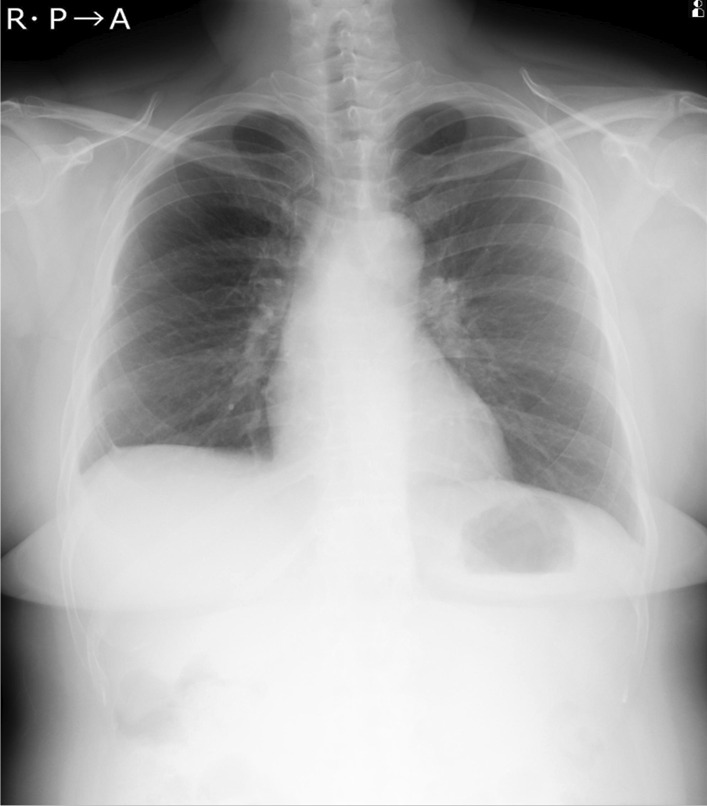
Chest X-ray at 6 months after administration of steroid showing a reduction in the upper mediastinal widening

In spite of the treatment for 3 weeks, skin rash appeared on her face, trunk and extremities, her dry cough and appetite loss worsened, and the computed tomography (CT) findings of lymphadenopathy did not improve, so these drugs were discontinued. Almost 6 weeks after surgical biopsy, it was clarified that no acid-fast bacilli were cultured in the liquid culture medium. Sarcoidosis was determined to be the appropriate diagnosis of these clinical symptoms, because mycobacterial infection had become less likely. The patient’s symptoms and radiological findings improved after oral administration of prednisolone 30 mg/day (0.5 mg/kg) (Figs. 4, 5). The dose was reduced by 5 mg every 2 weeks, and after 20 mg, the dose was gradually decreased by 5 mg every 3 months. At 6 months after the start of treatment, symptoms had completely disappeared, and the chest X-ray showed a marked reduction in the upper mediastinal widening (Fig. 6).

## Discussion

Here we report a case that required differentiation between sarcoidosis and disseminated MAC. The therapy for MAC was initiated on the basis of findings of epithelioid cell granuloma with marked necrosis, IgA antibody for MAC and radiological examinations, but response to the medication for MAC was poor, and no acid-fast bacilli were cultured 6 weeks after surgical biopsy. Thereafter, oral corticosteroid (30 mg/day) for sarcoidosis was administered, the patient’s symptoms and radiological findings dramatically improved, and no recurrence developed during the clinical course. Sarcoidosis can cause epithelioid cell granuloma with marked necrosis that is generally seen in infectious disease such as mycobacterial and fungal infections. Because the treatment for each disease is completely different, it is necessary to proceed with great care in differentiation, taking clinical, imaging and pathological findings into account.

Granulomatous inflammation is a histological pattern of tissue reaction which appears following cell injury [2]. Definitions employed range from inflammation characterized by the presence of lymphocytes, monocytes and plasma cells, to reactions identified by the presence of mononuclear phagocytes including epithelioid and giant cells [3]. Granulomatous lung disease is caused by various conditions. Differential diagnosis includes infectious, autoimmune, toxic, allergic, drug and neoplastic conditions [2, 4]. Therefore, the correlation of pathological findings with clinical, microbiological or radiological data is necessary to determine an etiology. Granulomas are characterized as necrotizing or non-necrotizing based on the presence or absence of necrosis [5]. In a study by Nazarullah *et al*., most cases of necrotizing granulomatous disease were found to have an infectious etiology, in particular mycobacterial and fungal infections [6].

Necrotizing granulomas are rarely seen in non-infectious disease. Sarcoidosis has been found to be the most frequent etiology in the non-infectious category and is characterized by the formation of non-necrotizing epithelioid cell granulomas [3]. By contrast, another report estimated that the ratio of necrosis in the granulomas of sarcoidosis is from 6 to 35% [1]. The mechanisms of necrosis have been variously described as fibrinoid, granular, eosinophilic granular and coagulative [1]. While necrosis in granulomas of sarcoidosis is usually small, spotty and inconspicuous, rare cases of sarcoidosis may exhibit larger and even confluent areas of necrosis in granulomas [1]. The most important differentiation is an infectious etiology such as *Mycobacterium* or fungi when there is necrosis in granulomas, because infection frequently causes granulomas with necrosis [2, 7]. Although glucocorticoids or methotrexate are administrated depending on the severity of disease in patients with sarcoidosis [8], these agents suppress the patient’s immunity. This can lead to a fatal state if the main etiology of disease is infection. Therefore, mycobacterial and fungal tests are essential to distinguish sarcoidosis from infectious disease. Although stains for acid-fast bacteria and fungi must always be undertaken, negative acid-fast stain does not exclude the possibility of mycobacterial infection. Among patients with pulmonary granulomatous inflammation categorized as mycobacterial etiology, acid-fast stain identified only 29% in tissue sections [6].

In the case in question, the differential diagnosis could mainly be divided into two, sarcoidosis and infection, in concordance with previous reports [7]. Although uveitis and high levels of ACE and sIL-2R suggested sarcoidosis, concern about infectious disease remained according to the necrotizing granuloma of pathological findings. It is reported that the etiology in necrotizing granulomatous lung disease with negative acid-fast bacteria and Gömöri methenamine silver staining is most likely infection, due to atypical mycobacteria [6]. The possibility of *Mycobacterium tuberculosis* was considered low because *Mycobacterium tuberculosis*-specific interferon-γ release assay was negative. The positive result of the IgA antibody for MAC suggested MAC infection, and CT findings of remarkable swelling of the mediastinum and hepatosplenomegaly was consistent with disseminated MAC. While disseminated MAC occurs mostly in immunocompromised hosts and involves systemic organs such as the spleen, lymph nodes, liver, intestine and bone marrow [9], several cases in immunocompetent hosts have been reported [9–11]. In the present case, initiation of therapy was required before the confirmed diagnosis because of the patient’s progressive symptoms of cough and consumption. It was feared that immunosuppressive therapy for sarcoidosis could worsen the patient’s condition if MAC infection were present. Therefore, we initially initiated medication for MAC infection including clarithromycin, rifampicin and ethambutol. However, there was a lack of response to treatment for MAC, and no acid-fast bacilli were cultured in the liquid culture medium in this case. Therefore, sarcoidosis was considered to be an appropriate diagnosis of these clinical conditions. Steroid therapy based on correcting the diagnosis led to improvement of the patient’s symptoms and radiological findings.

Necrotizing sarcoid granulomatosis (NSG) was considered as a differential diagnosis because of an epithelioid cell granuloma and the broad range of necrosis when mycobacterial infection was rejected. NSG is a rare systemic disease that is characterized by granulomatous angiitis, granuloma with necrosis and giant cell infiltrations [12]. This case lacked granulomatous vasculitis in the pathological findings. Therefore, the patient was diagnosed with sarcoidosis with marked necrosis. The pathogenesis of NSG is still unclear. In fact, there is the opinion that NSG should be classified as having the same spectrum as sarcoidosis, characterized by extensive granulomatous vasculitis [1].

## Conclusions

The most important differentiation is an infectious etiology, such as *Mycobacterium*, when there is necrosis in granulomas. However, it should be kept in mind that marked necrosis in granulomas occurs in patients with sarcoidosis. It is extremely important to carefully examine the clinical and pathological findings through discussion with the examining pathologist to reach the correct diagnosis.

## Data Availability

Data sharing is not applicable to this article as no data sets were generated or analyzed during this study.

## Abbreviations

ACE: Angiotensin-converting enzyme
CT: Computed tomography
MAC: *Mycobacterium avium* complex
sIL-2R: Soluble interleukin-2 receptor
